# Genome-wide identification of non-coding RNAs interacted with microRNAs in soybean

**DOI:** 10.3389/fpls.2014.00743

**Published:** 2014-12-23

**Authors:** Chu-Yu Ye, Hao Xu, Enhui Shen, Yang Liu, Yu Wang, Yifei Shen, Jie Qiu, Qian-Hao Zhu, Longjiang Fan

**Affiliations:** ^1^Department of Agronomy, Institute of Crop Sciences and Institute of Bioinformatics, College of Agriculture and Biotechnology, Zhejiang UniversityHangzhou, China; ^2^Guhe InformationHangzhou, China; ^3^Commonwealth Scientific and Industrial Research Organisation, Agriculture FlagshipCanberra, ACT, Australia

**Keywords:** miRNA, endogenous target mimics, phasiRNA, lipid metabolism, soybean

## Abstract

A wide range of RNA species interacting with microRNAs (miRNAs) form a complex gene regulation network and play vital roles in diverse biological processes. In this study, we performed a genome-wide identification of endogenous target mimics (eTMs) for miRNAs and phased-siRNA-producing loci (*PHAS*) in soybean with a focus on those involved in lipid metabolism. The results showed that a large number of eTMs and *PHAS* genes could be found in soybean. Additionally, we found that lipid metabolism related genes were potentially regulated by 28 miRNAs, and nine of them were potentially further regulated by a number of eTMs with expression evidence. Thirty-three miRNAs were found to trigger production of phasiRNAs from 49 *PHAS* genes, which were able to target lipid metabolism related genes. Degradome data supported miRNA- and/or phasiRNA-mediated cleavage of genes involved in lipid metabolism. Most eTMs for miRNAs involved in lipid metabolism and phasiRNAs targeting lipid metabolism related genes showed a tissue-specific expression pattern. Our bioinformatical evidences suggested that lipid metabolism in soybean is potentially regulated by a complex non-coding network, including miRNAs, eTMs, and phasiRNAs, and the results extended our knowledge on functions of non-coding RNAs.

## Introduction

Soybean (*Glycine max*) is one of the most important crops in the world. One of its main characteristics is its capacity to fix atmospheric nitrogen through symbioses with microorganisms (Schmutz et al., 2010). Soybean seeds contain abundant protein and oil that are crucial raw materials for food, feed, and other industrial applications (Lardizabal et al., 2008). Many microRNAs (miRNAs) with a potential role in stress responses (Zeng et al., 2010, 2012; Kulcheski et al., 2011; Li et al., 2011), nodulation (Subramanian et al., 2008; Wang et al., 2009; Li et al., 2010; Turner et al., 2012), and development (Joshi et al., 2010; Song et al., 2011; Wong et al., 2011; Shamimuzzaman and Vodkin, 2012) have been identified in soybean. miRNAs have also been implicated to play key roles in lipid metabolism of oil crops. For example, some miRNAs (e.g., miR156 and miR6029) from *Brassica napus*, another important edible oil crop, were differentially expressed in cultivars with different seed oil content or at different embryonic developmental stages (Zhao et al., 2012). Several studies also identified differentially expressed miRNAs in developing seeds of soybean, suggesting that these miRNAs might be involved in lipid metabolism (Song et al., 2011; Shamimuzzaman and Vodkin, 2012).

Small RNAs, including miRNA, repeat-associated small interfering RNA (ra-siRNA), *trans*-acting siRNA (tasiRNA), and natural antisense siRNA (nat-siRNA), play vital roles in plant development as well as in adaption to biotic and abiotic stresses (Jones-Rhoades et al., 2006; Rubio-Somoza and Weigel, 2011; Khraiwesh et al., 2012). miRNA regulates gene expression by mediating gene silencing at post-transcriptional level. miRNA is processed from primary miRNA (pri-miRNA) generated by RNA polymerase II. In plants, pri-miRNA is cleaved into miRNA precursor (pre-miRNA) containing a hairpin-like structure, which is further cleaved to give rise to a miRNA/miRNA^*^ duplex that is methylated at the 3′ ends. miRNA^*^ is generally degraded and the mature miRNA molecule is incorporated into a RNA-induced silencing complex to target complementary mRNAs through either cleavage or translational inhibition (Mallory and Vaucheret, 2006; Banks et al., 2012; Turner et al., 2012). In addition to silencing protein coding mRNAs, miRNAs are able to target *trans*-acting siRNA transcripts (*TAS*) to trigger production of phased tasiRNAs. On the other hand, the activity of miRNAs can be attenuated or abolished by endogenous target mimics (eTMs), which are usually non-coding transcripts and are able to sequester miRNAs in an uncleavable manner (Banks et al., 2012).

The first eTM identified in plants is the non-protein coding gene *INDUCED BY PHOSPHATE STARVATION1* (*IPS1*) from *Arabidopsis thaliana* (Franco-Zorrilla et al., 2007). *IPS1* binds to miR399, the phosphate starvation-induced miRNA. Because of a small loop caused by a few base pairs of mismatches at the expected miRNA cleavage site, *IPS1* RNA is not cleaved by miR399 but instead serving as a decoy for miR399 to interfere binding of miR399 to its canonical target, *PHO2*. Thus, plants overexpressing *IPS1* showed increased accumulation of the expression of miR-399 target *PHO2* and, concomitantly, reduced shoot Pi content. This mechanism of inhibition of miRNA activity is termed as target mimicry (Franco-Zorrilla et al., 2007). Several artificial target mimics (TMs) designed for different miRNAs with a similar paring pattern as that of *IPS1* and miR399 have been proven to affect the functions of their corresponding miRNAs in transgenic plants (Todesco et al., 2010; Ivashuta et al., 2011; Yan et al., 2012). Recently, eTMs of some conserved miRNAs have been computationally identified from intergenic regions or sequences with only short open reading frames in *A. thaliana*, *Oryza sativa*, and other plants (Banks et al., 2012; Wu et al., 2013). Additionally, *A. thaliana* transgenic plants overexpressing eTMs of miR160 and miR166 showed altered plant development, suggesting that these eTMs could be endogenously functional (Wu et al., 2013).

Biogenesis of tasiRNAs is triggered by interaction of miRNA at single or dual sites of the non-coding precursor *TAS* transcripts (Allen et al., 2005). Generation of tasiRNAs from *TAS* transcripts shows a phased pattern in which small RNAs are generated precisely in a head-to-tail arrangement starting from the miRNA cleavage site (Fei et al., 2013). It was found that many miRNAs could trigger the production of tasiRNAs, such as miR390, miR173, and miR828 in *A. thaliana* (Allen et al., 2005; Yoshikawa et al., 2005; Axtell et al., 2006; Rajagopalan et al., 2006). miR390 targets *TAS3* at two complementary sites to initiate production of tasiRNAs. miR173 targets both *TAS1* and *TAS2*, and miR828 directs tasiRNA biogenesis from *TAS4*. *TAS3* has been identified across a broad range of species (including moss, gymnosperms, and angiosperms), while *TAS1*, *TAS2*, and *TAS4* are found only in Arabidopsis and its close relatives (Fei et al., 2013; Hu et al., 2013). tasiRNAs mainly regulate the expression levels of their target transcripts in *trans*, e.g., *TAS3*-derived tasiARF targets members of the auxin response factor (ARF) family, including *ARF2*, *ARF3*, and *ARF4* (Williams et al., 2005). In addition to non-coding transcripts, miRNAs have also been shown to trigger production of phased siRNAs (phasiRNAs) from protein-coding loci, so called phasiRNA producing loci or *PHAS* loci, such as genes encoding pentatricopeptide repeat-containing proteins (PPRs), nucleotide-binding and leucine-rich-repeat-containing proteins (NB-LRRs), and MYB transcription factors in *Arabidopsis*, *Medicago*, *Malus*, *Prunus*, and *Nicotiana* (Howell et al., 2007; Zhai et al., 2011; Zhu et al., 2012; Xiao et al., 2014).

miRNAs interact with a diverse RNA species, such as protein coding target mRNAs and *PHAS* genes as well as eTMs, suggesting the presence of a complex gene regulation network involving miRNAs. In this study, we bioinformatically identified eTMs for miRNAs and *PHAS* genes targeted by miRNAs in soybean genome, and investigated their expression profiles in a wide range of organs and tissues. Our results suggest that a complex network including miRNAs, eTMs for miRNAs and phasiRNAs exists in soybean and it play important roles in diverse biological processes including lipid metabolism.

## Materials and methods

### Genomic, transcriptomic, and degradomic data sources

Genome sequences of *G. max* (version 1.0) and other four oil crops (or their progenitor species), *Ricinus communis* (v0.1), *Linum usitatissimum* (v1.0), *Brassica rapa* (v1.2), and *Gossypium raimondii* (v2.1), were obtained from Phytozome (http://www.phytozome.net/; v9.1) (Goodstein et al., 2012). Intergenic sequences of *G. max* were retrieved according to the information provided in the GFF file (Schmutz et al., 2010). Transcriptomic data (mRNA and small RNA) and degradomic data of *G. max* were downloaded from NCBI with the accession numbers listed in Supplementary Table S1 (Libault et al., 2010; Song et al., 2011, 2013; Shamimuzzaman and Vodkin, 2012; Collakova et al., 2013; Hu et al., 2013). *G. max* miRNAs were downloaded from miRBase (Release 20; http://www.mirbase.org/), and those with =4 and >4 mismatches compared to miRNAs of other plants in miRBase were considered as conserved miRNAs and soybean-specific miRNAs, respectively (Meyers et al., 2008).

### Identification of eTMs

Intergenic sequences of the *G. max* genome were collected as the eTM prediction library. eTM identification was performed using the bioinformatic pipeline previously described based on the following rules: (1) bulges composed of three nucleotides are only permitted at the positions corresponding to the 9th to 12th nucleotides counting from the 5′ end of a miRNA sequence; (2) perfect pairing is required from the 2nd to 8th positions at the 5′ end of a miRNA sequence; (3) the total number of mismatches and G/U pairs within the eTM and miRNA pairing region (excluding the central bulge) should be no more than three; and (4) the distance between an eTM and its upstream/downstream genes is longer than 200 nucleotides (Wu et al., 2013).

### Expression and conservation analysis of eTMs

To investigate the expression profiles of the predicted eTMs, publicly available RNA-Seq data generated from various tissues and organs of soybean (Table S1) were mapped to eTM sequences (including the miRNA binding sites and their 50-bp flanking regions) using TopHat (http://tophat.cbcb.umd.edu/; v2.0.9) with default settings. The abundance of RNA-seq reads aligned (at least three reads) to an eTM sequence indicates the expression level of the eTM. To investigate the conservation of the soybean eTMs in other oil crops or their progenitor species, including *R. communis*, *L. usitatissimum*, *B. rapa*, and *G. raimondii*, miRNA pairing sites of the *G. max* eTMs were used to Blast against the genomes of the four species (BlastN, 1e-1). Alignment of eTMs from soybean and other species was achieved using sequences including the miRNA pairing sites along with their flanking regions of the predicted eTMs (100 bp in total).

### Identification of putative PhasiRNA triggers

PhasiRNA-producing (*PHAS*) loci were identified in the intergenic regions of the *G. max* genome using the following algorithm previously described by Howell et al. (2007).

$$P=In\left[{\left(1+\sum_{i=1}^{8}k_{i}\right)}^{n-2}\right],P>0$$

(where *n* = number of phase cycle positions occupied by at least one small RNA read within an eight-cycle window, and *k* = total number of reads for all small RNAs falling into a given phase within an eight-cycle window). In this study, all phasiRNA-producing loci were considered as *PHAS* loci, although they were located in the non-coding regions of the *G. max* genome. For a miRNA to be considered as a putative phasiRNA trigger, (1) it has to be able to bind to a *PHAS* locus within the regions generating phased siRNAs or their flanking 200-nt regions; (2) the putative miRNA cleavage site (corresponding to the position between the 10th and 11th nucleotides counting from the 5′ end of a miRNA) should be at a position corresponding to a phase register. (Liu et al., 2013) The expression level of a *PHAS* gene was measured by the abundance of siRNA reads (RPM) aligned to all phases of the *PHAS* locus.

### Analysis of potential genes related to lipid biosynthesis in soybean

The lipid biosynthesis related genes in Arabidopsis were downloaded from http://aralip.plantbiology.msu.edu/ and were then searched against the *G. max* genome to find their homologous soybean genes using BlastP with an *E*-value <1e-5 and identity >50% (Li-Beisson et al., 2013; Wang et al., 2014).

### Target prediction and validation by degradomic data

Targets of soybean miRNAs were predicted by search against all annotated transcripts of *G. max* using psRNATarget (http://plantgrn.noble.org/psRNATarget/) with the default settings (maximum expectation: 3.0; length for complementary scoring: 20 bp; target accessibility-allowed maximum energy to unpair the target site: 25.0; flanking length around target site for target accessibility analysis: 17 bp in upstream and 13 bp in downstream; range of central mismatch leading to translational inhibition: 9–11 nt) (Dai and Zhao, 2011). These settings, except for maximum expectation that was set as 1 to reduce the false positive prediction rate, were also used in prediction of targets of phasiRNAs. To make the miRNA target prediction more accurate, psRobot was also used with the score of three (Wu et al., 2012). The same results obtained from these two programs were adopted. The soybean homologs of Arabidopsis genes related to lipid biosynthesis that were predicted to be targets of miRNAs or phasiRNAs were considered as candidates involved in lipid biosynthesis and regulated by miRNAs or phasiRNAs in soybean. Publicly available degradome sequencing data (Table S1) were used to validate the predicted targets. A predicted target was considered as cleaved by a miRNA or phasiRNA if the predicted cleavage site had degradome reads perfectly aligned to, i.e., the 5′ ends of degradome reads were exactly aligned with the predicted cleavage site of a miRNA or phasiRNA.

## Results

### Identification of endogenous target mimics

**Figure 1:**
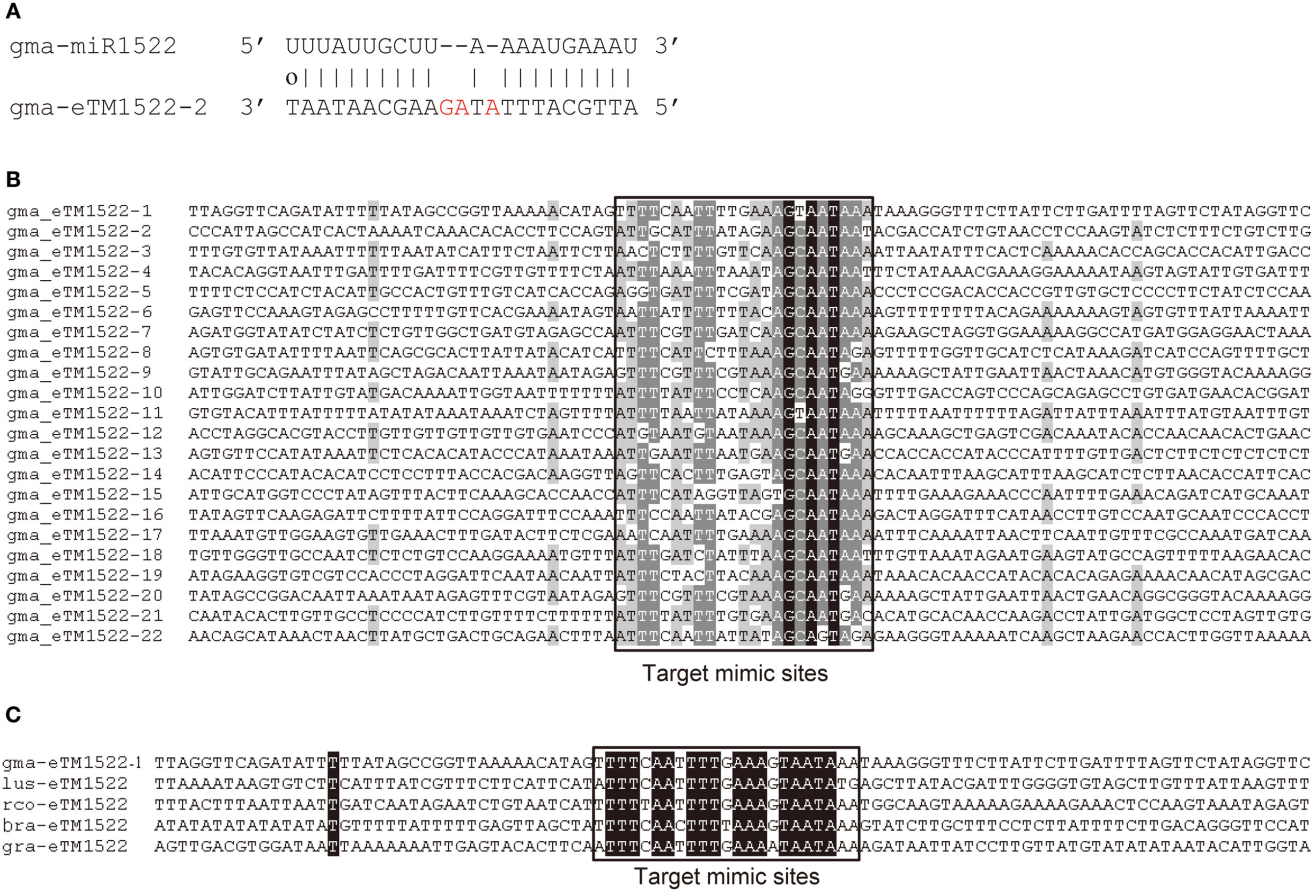
**Endogenous target mimics (eTMs) for gma-miR1522 in soybean. (A)** The predicted base-pairing pattern between gma-miR1522 and one of its eTMs; **(B)** Sequence alignment of the 22 expressed eTMs for gma-miR1522; **(C)** Sequence alignment of eTMs for miR1522 in *G. max* and other four species (lus: *L. usitatissimum*; rco: *R. communis*; bra: *B. rapa*; and gra: *G. raimondii*).

Intergenic non-coding sequences in the soybean genome (Williams 82) were selected to predict putative eTMs for all 554 soybean miRNAs. Of the 554 soybean miRNAs, 334 (60.3%) had eTMs predicted. In total, we predicted 10,410 eTMs for 144 conserved miRNAs and 32,555 for 190 soybean-specific miRNAs (Table S2). As an example, sequence alignment between the soybean-specific gma-miR1522 and one of its eTMs, gma-eTM1522-2, was shown in Figure 1A, which showed a typical pairing pattern between a miRNA and its eTM, i.e., three unmatched nucleotides in the eTM at the region corresponding to the 10th to 12th positions from the 5′ end of gma-miR1522 (Figure 1A). A number of eTMs were predicted for gma-miR1522 and 22 of them were expressed in at least one of the tissues or organs examined in this study (Tables S1, S2). Sequence alignment of these 22 expressed eTMs showed that they were conserved only in the region corresponding to the predicted miRNA binding sites (i.e., the target mimic sites) (Figure 1B). Sequences containing a well conserved target mimic site for gma-miR1522 were also found in the four additional species (*R. communis*, *L. usitatissimum*, *B. rapa*, and *G. raimondii*) examined in this study. Similarly, only the target mimic sites but not their flanking regions were conserved in these predicted eTMs (Figure 1C), consistent with the observation previously reported (Wu et al., 2013). Prediction of conserved eTMs for gma-miR1522 in all four species examined in this study suggests that miR1522 could be present, although it has not been reported, in these species.

Expression of the predicted eTMs is a prerequisite for them to be functional. To examine whether the predicted eTMs are expressed in soybean, we analyzed 20 published RNA-Seq datasets generated from a wide range of organs or tissues, including vegetative and reproductive tissues as well as developing seeds (Table S1). In total, 457 eTMs for 126 miRNAs (144 eTMs for 61 conserved miRNAs and 313 for 65 soybean-specific miRNAs) were found to be expressed in at least one of the examined tissues (Table 1, Table S2). These 457 eTMs for miRNAs with high confidence were used for our further investigation. Of these 457 expressed eTMs, 285 had their homologs found in at least one of the other four species examined in this study. For example, the homolog of eTM1522 could be found in the all four species (Figure 1C). To rule out the possibility that the expressed eTMs are potential miRNA targets, we analyzed seven published degradome datasets and found that no degradome read was assigned to the miRNA pairing regions of the 457 eTMs, demonstrating no miRNA-mediated cleavage in these eTMs and suggesting that they could function as mRNA decoys. To know the potential functionality of these eTMs, we performed Gene Ontology (GO) analysis for the predicted targets of the 126 miRNAs with expressed eTMs. As expected these miRNA targets were found to be involved in a wide range of biological progresses (Table S3); we thus expect that the expressed eTMs could also play a role in the related biological progresses. Taken together, the above results suggest that the functions of a number of miRNAs were potentially regulated by eTMs in soybean.

**Table 1:** **Number of miRNAs and their partners (eTMs and *PHAS* loci) in soybean**.

**Type of miRNAs**	**Number of miRNAs (lipid^a^)**	**Number of expressed eTMs for miRNAs (lipid^b^)**	**Number of *PHAS* loci with a miRNA trigger identified (lipid^c^)**
Conserved	281 (45)	144 (37)	157 (3)
Soybean-specific	273 (52)	313 (82)	827 (46)
Total	554 (97)	457 (119)	984 (49)

a Number of miRNAs that were predicted to target lipid biosynthesis related genes.

b Number of eTMs for miRNAs that were predicted to target lipid biosynthesis related genes.

c Number of PHAS loci producing phasiRNAs that were predicted to target lipid biosynthesis related genes.

### eTMs for miRNAs targeting genes related to lipid metabolism

To investigate a role of miRNAs and their eTMs in lipid metabolism in soybean, we first identified soybean homologs of Arabidopsis genes involved in lipid metabolism. This analysis found 1507 genes with a potential role in lipid metabolism in soybean. We then predicted potential targets of all soybean miRNAs (in total 554, including 281 conserved and 273 soybean-specific miRNAs) downloaded from miRBase (Release 20). Based on these two analyses, we found that 89 soybean homologs of Arabidopsis genes involved in lipid biosynthesis were predicted targets of 97 miRNAs (45 conserved and 52 soybean-specific miRNAs) (Table 1). Using the publicly available degradome data we found that 25 of these genes were cleaved by miRNAs from 18 miRNA families (Table S4, Figure S1). According to gene annotation, these genes are involved in a diverse pathways related to lipid metabolism, such as fatty acid synthesis, elongation and degradation as well as lipid trafficking.

**Figure 2:**
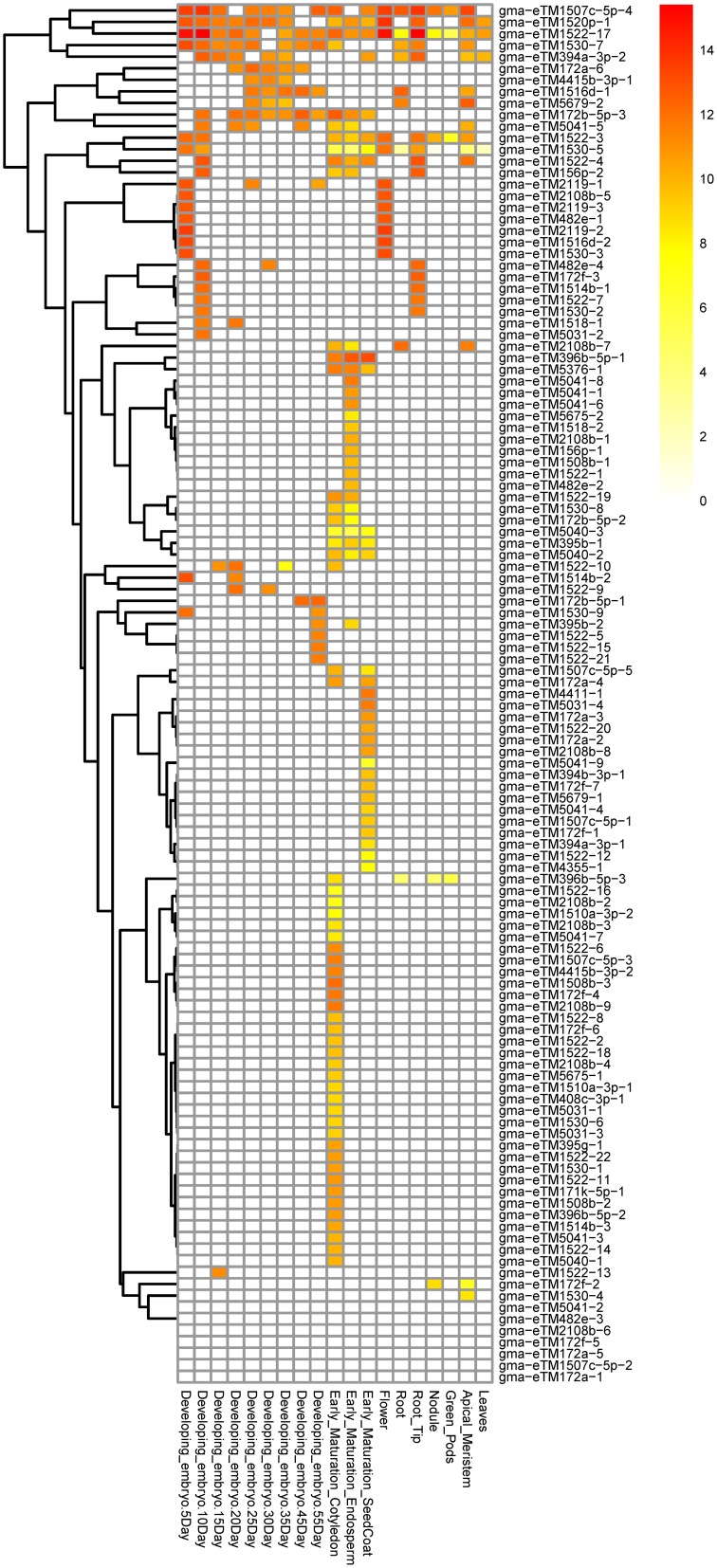
**Expression patterns of the eTMs for miRNAs with a potential role in lipid biosynthesis**. Expression values were log_2_-transformed.

Of the 457 expressed eTMs, 119 were eTMs for 33 miRNAs predicted to target genes involved in lipid metabolism, including 37 eTMs for 14 conserved miRNAs and 82 eTMs for 19 soybean-specific miRNAs (Tables S5, S6). Some of these eTMs were expressed in most of the tissues or organs examined whereas the majority of these eTMs were specifically expressed in certain tissue(s). For example, gma-eTM1522-17 seemed to be universally expressed while expression of 33, 16 and 10 eTMs was only found in cotyledon, seed coat and endosperm at the early stage of seed maturation, respectively (Figure 2). Some miRNAs had multiple expressed eTMs that showed differential expression pattern in different tissues. For instance, we identified 22 expressed eTMs for gma-miR1522, which was predicted to target a gene encoding ABC transporter (Glyma03g29150, homolog of At3g21090 that is required for lipid transport; Pighin et al., 2004; Table S5). Four of the 22 eTMs were expressed in four or more different samples shown in Figure 2, whereas 15 of the 22 eTMs were only expressed in one of the samples (Figure 2). Differential expression of different eTM members for the same miRNA was also observed for eTMs of other miRNAs, such as gma-miR1530 (10 eTMs) and gma-miR504 (9 eTMs), which were predicted to target a phosphatidylinositol kinase gene (Glyma04g39150, homolog of At5g64070 that is involved in phospholipid signaling; Li-Beisson et al., 2013) and genes (Glyma04g06630 and Glyma06g06720) related to triacylglycerol biosynthesis (Table S5), respectively. Additionally, 65 of the 119 expressed eTMs for miRNAs predicted to target genes involved in lipid metabolism had a homolog in at least one of the other four species used in this study (Table S7; eTMs for miR1522 as an example was shown in Figure 1C). The conservation of eTMs further supported the potential functionality of these eTMs in lipid metabolism.

### Identification of miRNA-mediated PhasiRNA-producing loci

**Figure 3:**
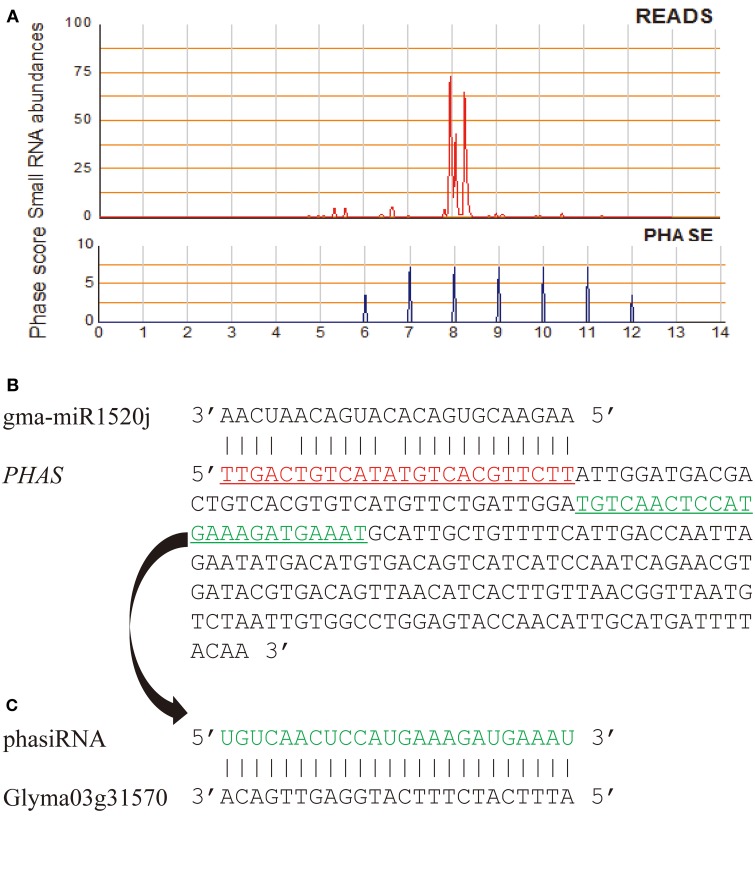
**A *PHAS* locus (Gm17:18214876… 18215211) targeted by gma-miR1520j. (A)** The siRNA abundance and phasing score of the phased siRNAs generated from the *PHAS* locus *PHAS1520j-3*; **(B)** Sequence segment of *PHAS1520j-3*. The gma-miR1520j binding site is aligned with gma-miR1520j (predicted target location: Gm17:18214862… 18214885); **(C)** Sequence alignment between a phasiRNA (Gm17: 18215043… 18215066) derived from *PHAS1520j-3* locus and its predicted target (Glyma03g31570, a gene encoding acylhydrolase involved in oxylipin metabolism).

PhasiRNA-producing (*PHAS*) loci of the *G. max* (Williams 82) genome were identified based on small RNA reads from eight small RNA libraries (Table S1) and the algorithm described by Howell et al. (2007). A large number of *PHAS* loci generating 21-nt or 24-nt phasiRNAs were detected in the *G. max* genome (phase score >1.4). Even when using a stringent phase score (phase score >20), 1573 of 21-nt and 4681 of 24-nt *PHAS* loci could still be identified (Table S8). Of these *PHAS* loci, 984 (262 of 21-nt and 722 of 24-nt *PHAS* loci) had at least a miRNA binding site predicted within the regions generating phased siRNAs and/or their flanking 200-nt regions (Table S9). Some of the identified phasiRNA triggers have been previously identified in other studies, such as miR390, miR156, miR2118, miR393, miR1508, miR1510, and miR1514 (Zhai et al., 2011; Hu et al., 2013). Of the 984 *PHAS* loci, 157 and 827 were triggered by conserved and soybean-specific miRNAs, respectively, and phasiRNAs from 49 such *PHAS* loci were predicted to target genes related to lipid metabolism (Table 1, Tables S5, S9). For example, gma-miR1520j was predicted to trigger production of 24-nt phasiRNAs from a locus (*PHAS1520j-3*) located in soybean chromosome 17, and a phasiRNA from this locus was predicted to target Glyma03g31570, which encodes an acylhydrolase involved in oxylipin metabolism (Figure 3). Of these 49 *PHAS* genes, 12 were cleaved by their miRNA triggers according to degradome data (Table S9).

**Figure 4:**
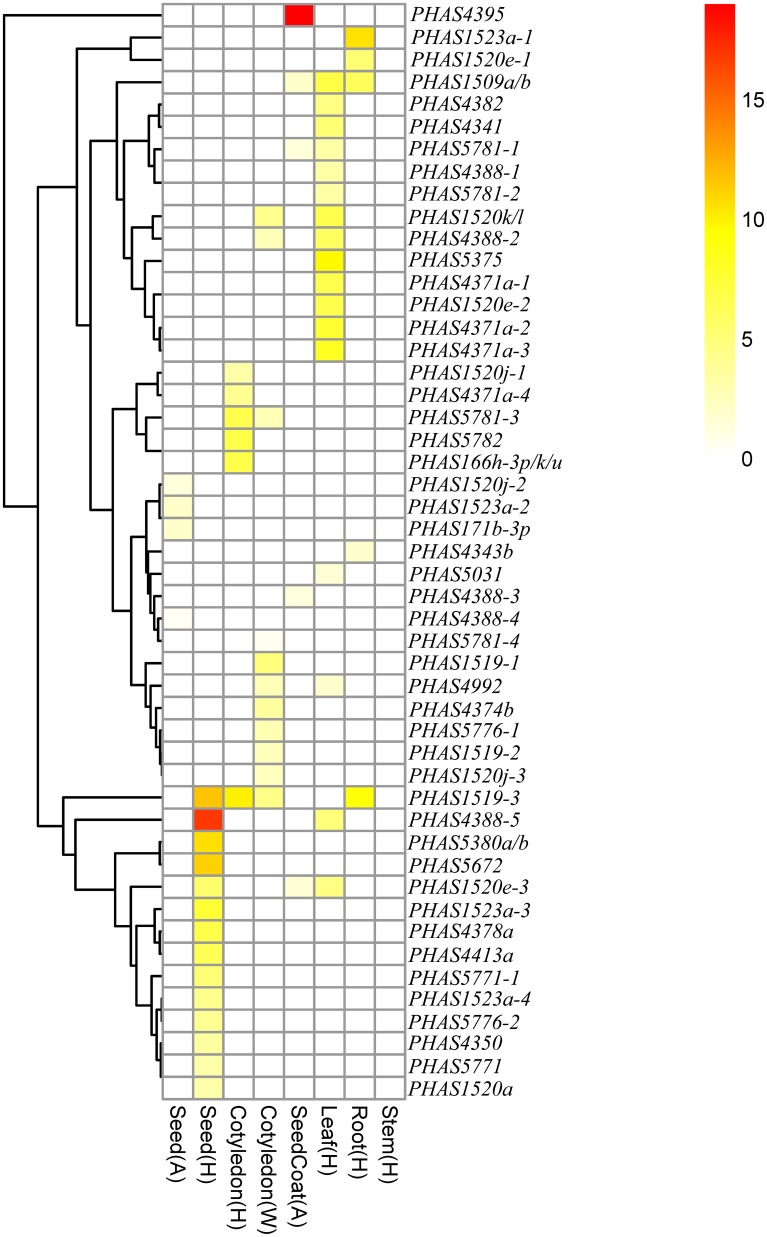
**Expression patterns of the *PHAS* genes producing phasiRNAs that were predicted to target lipid biosynthesis related genes in soybean**.

The expression levels of the 49 *PHAS* genes producing phasiRNAs targeting genes related to lipid metabolism were investigated based on published small RNA data (Figure 4, Tables S1, S10). Of the 49 *PHAS* genes, nine seemed to be expressed in two or more of the eight tissue samples shown in Figure 4, although the expression levels were low in most samples. One exception was the *PHAS4388-5* gene, which was targeted by gma-miR4388 to produce phasiRNA targeting Glyma12g28470 that encodes an acyltransferase. This *PHAS* gene was highly expressed in seed but lowly expressed in leaf in cultivar Heinong44 (Figure 4). Interestingly, none of the 49 *PHAS* genes seemed to be expressed in stems of soybean although some of these *PHAS* genes were relatively highly expressed in roots and/or leaves.

## Discussion

### A large number of eTMs and *PHAS* genes in soybean

It has become clear that numerous non-coding RNA transcripts interact with miRNAs and are part of the network regulating development and stress responses in both plants and animals (Tay et al., 2014). Because of their ability to sequester miRNAs away from their cleavable targets, RNA molecules with miRNA binding sites but un-cleavable by miRNAs have been reported with different terminology, such as “miRNA sponges/decoys,” “endogenous target mimics” or “competing endogenous RNA” (Banks et al., 2012; Wu et al., 2013). In this study, we found that the functionalities of a large number of miRNAs could be potentially regulated by eTMs in soybean. Our results together with previously published results further suggest that eTMs could be widespread regulators of miRNA functions in plants (Wu et al., 2013). Among the 42,965 computationally predicted eTMs in the soybean genome, only 457 eTMs were found to be expressed. It was partly due to that the RNA-Seq data we used only included samples from different tissues and developmental stages of soybean. The functions of eTMs are related to their corresponding miRNAs, many of which function in stress responses. Therefore, RNA-Seq data from samples with stress treatment should provide expression evidence for more eTMs. However, the huge discrepancy between computationally-predicted eTMs and those with expression support also demonstrated the limitation of the computational prediction for eTMs. The expression evidence is thus important for the identification of authentic eTMs. Another kind of interaction between miRNAs and non-coding RNAs involves generating phasiRNAs from the miRNA-targeted non-coding RNAs. The well-known phasiRNAs generated from miRNA-targeted non-coding RNAs are tasiRNAs. *TAS* gene has been demonstrated to play a role in diverse biological processes, such as development by targets auxin response factor (ARF) and MYB protein family and disease resistance by nucleotide binding leucine-rich repeat NB-LRR (Williams et al., 2005; Zhai et al., 2011). In addition to *TAS* genes, we showed here that numerous intergenic regions in the soybean genome are targeted by miRNAs to produce phasiRNAs, which are able to further target other mRNAs. This result suggested that miRNA-mediated production of phasiRNAs may have a much more profound effect on biological processes in soybean than we previously thought.

More eTMs (32,555 vs. 10,410 eTMs based on computational prediction; 313 vs. 144 eTMs with expression support) were found for the 190 soybean-specific miRNAs than for the 144 conserved miRNAs. Similarly, we also found that soybean *PHAS* genes are more likely to be targeted by soybean-specific miRNAs than by conserved miRNAs (827 vs. 157 *PHAS* genes). This phenomenon may suggest that eTMs and phasiRNAs are two types of important regulators co-evolved with soybean-specific miRNAs to regulate soybean-specific biological processes, and that the co-evolution of the non-coding RNA network in soybean may be a result of species-specific adaptations. In addition, we found that some biological processes were significantly enriched in the predicted target genes of miRNAs that have eTMs with expression evidence, suggesting a specified role of the interaction between miRNAs and their eTMs in these biological processes (Table S3). For example, the genes with GO categories related to biological regulation were significantly enriched, indicating the role of eTMs in gene regulation through the network involved miRNAs. Some genes related to stress response and cell death were significantly enriched, suggesting that eTMs might play key roles in these two biological processes through interaction with miRNAs.

### The non-coding RNA network involved in lipid metabolism in soybean

**Figure 5:**
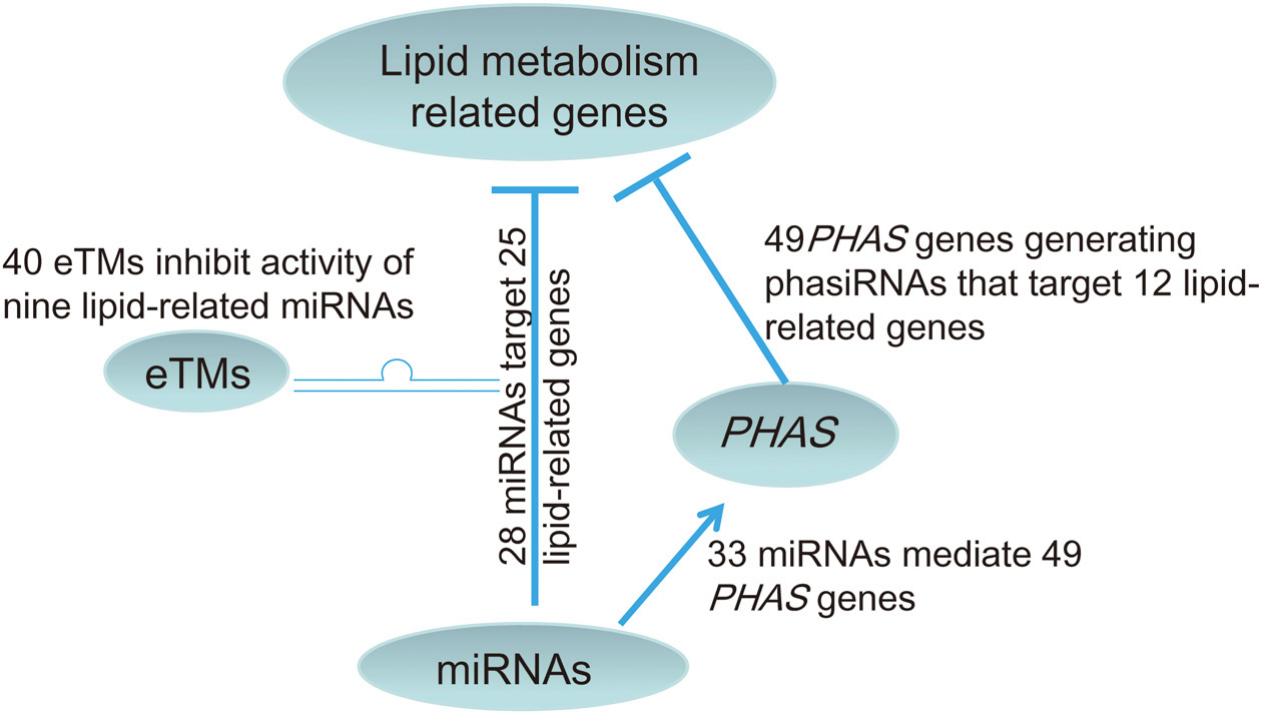
**A model for the non-coding RNA network involved in lipid biosynthesis in soybean**. The network contains 28 miRNAs directly targeting lipid biosynthesis related genes with degradome data support and 33 miRNAs predicted to target 49 *PHAS* loci that produced phasiRNAs to target 12 lipid biosynthesis related genes. Of the 28 miRNAs directly targeting lipid biosynthesis related genes, nine have predicted eTMs supported by expression data.

At least 120 enzymatic reactions and more than 600 genes are involved in the 24 pathways related to Acyl-lipid metabolism in Arabidopsis (http://aralip.plantbiology.msu.edu/) (Li-Beisson et al., 2013). Although the pathways and protein coding genes associated with lipid biosynthesis in higher plants have been largely uncovered, the roles of the non-coding RNAs in lipid biosynthesis are still poorly understood. In this study, we investigated the potential roles of the non-coding RNA network, including miRNAs and their partners, i.e., eTMs and miRNA-triggered *PHAS* genes, in lipid metabolism in soybean. Understanding the functions of the miRNA network in regulating lipid metabolism in soybean will be of great value for the cultivation of soybean cultivars with increased oil content. Overall, our results revealed that lipid metabolism related genes in soybean are potentially directly regulated by 28 miRNAs with degradome data support, and that nine of these 28 miRNAs are potentially further regulated by a number of eTMs with 40 of them supported by expression data. The lipid metabolism pathways regulated by the network involving these nine miRNAs and 40 eTMs includes fatty acid synthesis, elongation, degradation, oxylipin metabolism, and phospholipid signaling (Table S5). In addition, 33 miRNAs were found to trigger production of phasiRNAs from 49 *PHAS* genes, which were able to target lipid biosynthesis related genes (Figure 5). We found that the lipid metabolism related genes potentially regulated by miRNAs and their partners in soybean were Arabidopsis homologs involved in 23 of the 24 pathways related to Acyl-lipid metabolism (Table S5). These results provided bioinformatical evidences for the hypothesis that lipid metabolism in soybean is regulated by a complex non-coding RNA network including miRNAs, eTMs and phasiRNAs.

This hypothesis was supported by several pieces of evidence. Firstly, of the 97 miRNAs predicted to target lipid biosynthesis related genes, 28 were confirmed to cleave their targets based on the data from seven publicly available degradome libraries (Table S4). Secondly, 119 eTMs for miRNAs predicted to target genes related to lipid metabolism were found to be expressed and most of them had a tissue-specific expression pattern (Figure 2); 55% of them are evolutionarily conserved in their target mimic sites in the other four species examined in this study. Thirdly, majority of the 49 *PHAS* genes generating phasiRNAs that were predicted to target lipid biosynthesis related genes also showed a tissue-specific expression pattern (Figure 4), and miRNA-mediated cleavage evidence was found for 12 of the 49 *PHAS* genes based on the publicly available degradome data (Table S9). Fourthly, some of the genes related to lipid metabolism might be regulated by only one component of the network but some could be controlled by a cascade of the network or even all three types of non-coding RNAs, i.e., miRNA, eTM and phasiRNA. Of the 18 miRNA families targeting lipid biosynthesis genes that were validated by degradome data, at least three (gma-miR1520j, gma-miR4388 and gma-miR4992) were also able to target *PHAS* genes to produce phasiRNAs that in turn to target genes involved in lipid metabolism (Tables S4, S9). Furthermore, at least nine (gma-miR1508b, gma-miR1520p, gma-miR1530, gma-miR2108b, gma-miR394a-3p, gma-miR395b, gma-miR396b-5p, gma-miR5041, and gma-miR5769) of the 18 miRNA families had expressed eTMs (Table S2), suggesting that the functionality of these miRNAs could be attenuated in certain tissues.

In this study, our focus was on non-coding RNAs interacting with miRNAs in soybean; however, in addition to non-coding RNAs, our analyses also found that 120 protein-coding genes were potentially targeted by miRNAs to generate phasiRNAs (Table S9). Additionally, we only used intergenic regions in eTM prediction in this study. In fact, protein-coding, intronic, and antisense sequences all could function as eTMs (Ponting et al., 2009; Wu et al., 2013). Furthermore, it is our further interest to know whether plants contain other kinds of RNA molecules, such as circular RNAs and pseudogene competing endogenous RNAs reported in humans and animals (Tay et al., 2014), and whether they interact with miRNAs to regulate biological processes in plants.

## Author contributions

Longjiang Fan conceived and designed the study. Chu-Yu Ye, Hao Xu, Enhui Shen, Yang Liu, Yu Wang, Yifei Shen, and Jie Qiu analyzed the data. Chu-Yu Ye, Qian-Hao Zhu, and Longjiang Fan wrote the paper.

### Conflict of interest statement

The authors declare that the research was conducted in the absence of any commercial or financial relationships that could be construed as a potential conflict of interest.
