# Supplementary material for: Genome-wide identification of non-coding RNAs interacted with microRNAs in soybean
Source: Front Plant Sci. 2014 Dec 23;5:743. doi: 10.3389/fpls.2014.00743 (PMC4274897; doi:10.3389/fpls.2014.00743)
Supplement: Supplementary file 1 [file Presentation1.PPTX]

## Slide 1
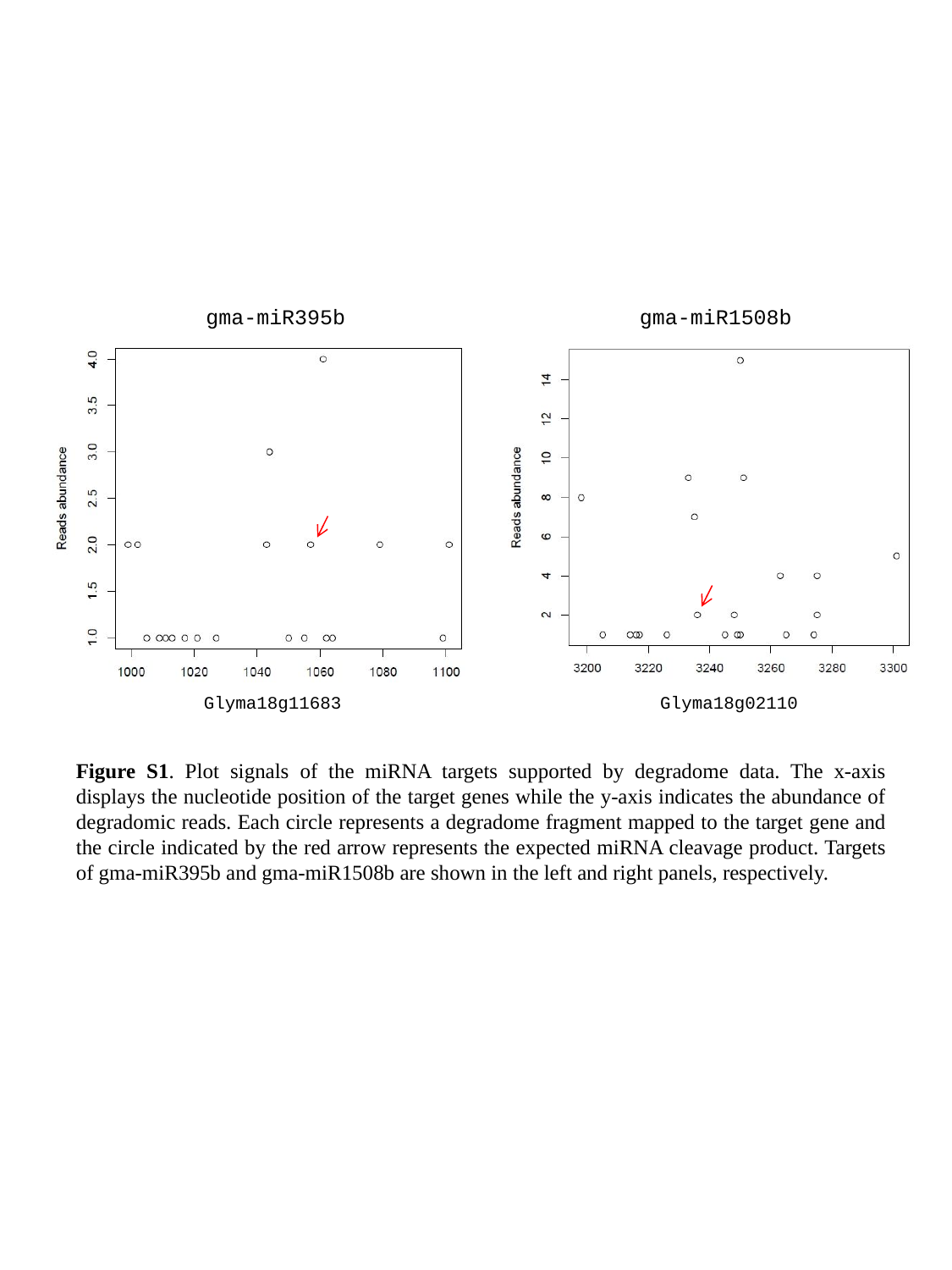

gma-miR395b
gma-miR1508b
Glyma18g11683
Glyma18g02110
Figure S1. Plot signals of the miRNA targets supported by degradome data. The x-axis displays the nucleotide position of the target genes while the y-axis indicates the abundance of degradomic reads. Each circle represents a degradome fragment mapped to the target gene and the circle indicated by the red arrow represents the expected miRNA cleavage product. Targets of gma-miR395b and gma-miR1508b are shown in the left and right panels, respectively.
